# Lymph node examination and survival in resected pancreatic ductal adenocarcinoma: retrospective study

**DOI:** 10.1093/bjsopen/zrad125

**Published:** 2024-01-25

**Authors:** Ruediger Goess, Carsten Jäger, Julie Perinel, Ilaria Pergolini, Elke Demir, Okan Safak, Florian Scheufele, Stephan Schorn, Alexander Muckenhuber, Mustapha Adham, Alexander Novotny, Güralp O Ceyhan, Helmut Friess, Ihsan Ekin Demir

**Affiliations:** Department of Surgery, Klinikum rechts der Isar, Technical University of Munich, School of Medicine, Munich, Germany; German Cancer Consortium (DKTK), Partner Site Munich, Munich, Germany; CRC 1321 Modelling and Targeting Pancreatic Cancer, Munich, Germany; Department of Surgery, Klinikum rechts der Isar, Technical University of Munich, School of Medicine, Munich, Germany; German Cancer Consortium (DKTK), Partner Site Munich, Munich, Germany; CRC 1321 Modelling and Targeting Pancreatic Cancer, Munich, Germany; Department of Digestive Surgery, E. Herriot Hospital, Hospices civils de Lyon, Lyon, France; Department of Surgery, Klinikum rechts der Isar, Technical University of Munich, School of Medicine, Munich, Germany; German Cancer Consortium (DKTK), Partner Site Munich, Munich, Germany; CRC 1321 Modelling and Targeting Pancreatic Cancer, Munich, Germany; Department of Surgery, Klinikum rechts der Isar, Technical University of Munich, School of Medicine, Munich, Germany; German Cancer Consortium (DKTK), Partner Site Munich, Munich, Germany; CRC 1321 Modelling and Targeting Pancreatic Cancer, Munich, Germany; Department of Surgery, Klinikum rechts der Isar, Technical University of Munich, School of Medicine, Munich, Germany; German Cancer Consortium (DKTK), Partner Site Munich, Munich, Germany; CRC 1321 Modelling and Targeting Pancreatic Cancer, Munich, Germany; Department of Surgery, Klinikum rechts der Isar, Technical University of Munich, School of Medicine, Munich, Germany; German Cancer Consortium (DKTK), Partner Site Munich, Munich, Germany; CRC 1321 Modelling and Targeting Pancreatic Cancer, Munich, Germany; Department of Surgery, Klinikum rechts der Isar, Technical University of Munich, School of Medicine, Munich, Germany; German Cancer Consortium (DKTK), Partner Site Munich, Munich, Germany; CRC 1321 Modelling and Targeting Pancreatic Cancer, Munich, Germany; Institute of Pathology, Klinikum rechts der Isar, Technical University of Munich, School of Medicine, Munich, Germany; Department of Digestive Surgery, E. Herriot Hospital, Hospices civils de Lyon, Lyon, France; Department of Surgery, Klinikum rechts der Isar, Technical University of Munich, School of Medicine, Munich, Germany; Department of General Surgery, HPB-Unit, School of Medicine, Acibadem Mehmet Ali Aydinlar University, Istanbul, Turkey; Department of Surgery, Klinikum rechts der Isar, Technical University of Munich, School of Medicine, Munich, Germany; German Cancer Consortium (DKTK), Partner Site Munich, Munich, Germany; CRC 1321 Modelling and Targeting Pancreatic Cancer, Munich, Germany; Department of Surgery, Klinikum rechts der Isar, Technical University of Munich, School of Medicine, Munich, Germany; German Cancer Consortium (DKTK), Partner Site Munich, Munich, Germany; CRC 1321 Modelling and Targeting Pancreatic Cancer, Munich, Germany; Department of General Surgery, HPB-Unit, School of Medicine, Acibadem Mehmet Ali Aydinlar University, Istanbul, Turkey; Else Kröner Clinician Scientist Professorship for Translational Pancreatic Surgery, Munich, Germany

## Abstract

**Background:**

The minimum number of examined lymph nodes (ELN) required for adequate staging and best prediction of survival has not been established in pancreatic ductal adenocarcinoma (PDAC). The aim of the study was to investigate the influence of ELN on staging and survival in PDAC.

**Methods:**

Patients undergoing partial or total pancreatectomy for PDAC at two European university hospitals between 2007 and 2018 were retrospectively reviewed. Multivariate Cox regression model and survival analyses were performed to verify adequate staging.

**Results:**

Overall 341 (73 per cent) patients showed lymph node metastasis (N1/N2), whereas 125 (27 per cent) patients had no lymph node involvement (N0). With increasing number of ELN, the proportion of positive lymph nodes increased. The minimum number of ELN needed to detect lymph node involvement was 21. In multivariate analysis, examination of <21 lymph nodes was a significant negative predictor for survival. Examination of ≥21 ELN reversed this effect and ruled out possible misclassification.

**Conclusion:**

The number of ELN affects survival in PDAC. Possible misclassification was identified when <21 lymph nodes were examined. Therefore, at least 21 lymph nodes must be examined to avoid false lymph node classification in all types of resection.

## Teaser

Four hundred and sixty-six patients undergoing pancreatectomy for pancreatic ductal adenocarcinoma (PDAC) at two European university hospitals were analysed to determine the minimum number of examined lymph nodes required for adequate staging. To avoid nodal misclassification and improve survival in the long term at least 21 lymph nodes must be examined in PDAC.

## Introduction

Pancreatic ductal adenocarcinoma is an early metastatic tumour and remains one of the most lethal malignancies^1,2^. Surgical resection with lymphadenectomy provides the only chance for cure. Lymph node (LN) metastases are common and appear in around 60–90 per cent of all resected patients^3,4^. In node-negative patients, a median survival of 25–30 months can be achieved, whereas in node-positive patients the median survival decreases to around 15 months^4–6^. Several studies have demonstrated that the number of positive lymph nodes influences survival^3,7,5^.

Various randomized controlled trials showed that extended lymphadenectomy does not generate any survival benefit compared to standard regional lymphadenectomy^8–10^. The number of examined lymph nodes (ELN) might particularly be influenced by variations in lymph node dissection, individual surgeon skills, the gross pathologist expertise, the anatomic constitution of each patient, and neoadjuvant treatment. With the standardization of operation technique, the variation in the number of ELN should theoretically not be pronounced. Due to the fact that not all resected lymph nodes are always examined, it could lead to a misclassification of N status because positive LN can be overlooked. It was also shown that the number of ELN and not only the involved LN is a prognostic factor for survival in patients after distal pancreatectomy^11^.

There is no clear consensus on the minimum number of ELN needed for adequate staging in pancreatic resections for pancreatic ductal adenocarcinoma (PDAC). Several studies reported numbers varying between 11 and 28 required lymph nodes^3,5,11–16^.

The aim of the current study was to investigate the influence of ELN on staging and survival in all different types of pancreatic resection and to identify the minimum number of ELN needed for the best prediction of survival in pancreatic cancer.

## Methods

### Study design and patient selection

Patients with histologically confirmed PDAC who underwent elective curative tumour resection with regional lymphadenectomy at the Department of Surgery, Klinikum rechts der Isar, Munich, Germany or the Department of Digestive Surgery, E. Herriot Hospital, Hospices civils de Lyon, France between 2007 and 2018 were included in the study. All patients were identified from a prospectively maintained pancreatic cancer database containing standard demographic, clinical, operative and pathological data. The institutional review board (IRB) approved the prospective data collection with retrospective data review for the study (Ethics Committee Approval No.: 2022-438-S-NP).

**Fig. 1:**
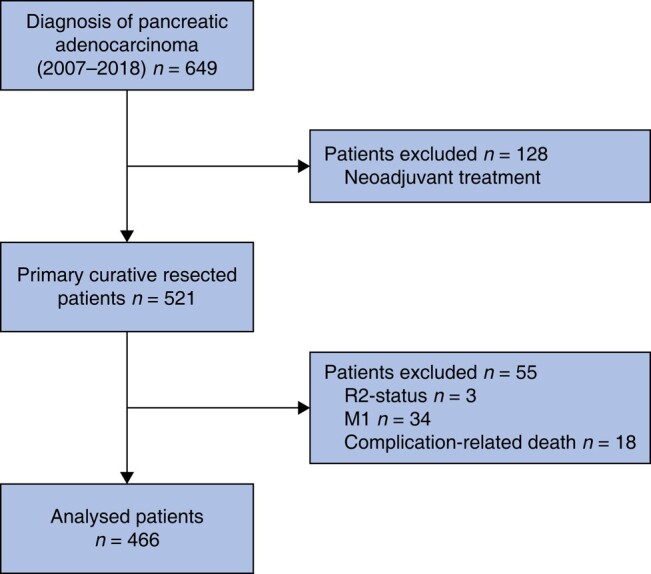
Flowchart detailing inclusion and exclusion criteria of the final study cohort

In all patients, a primary curative resection was performed depending on the localization of the tumour, that is pancreaticoduodenectomy (PD), distal pancreatectomy (DP), or total pancreatectomy (TP) with standard lymphadenectomy. Patients with organ metastasis diagnosed during resection (M1), macroscopically incomplete resection (R2 status), neoadjuvant treatment and complication-related postoperative death (≤30 days) were excluded from the study for avoiding bias in the survival analyses (*Fig. 1*). R0 status was defined as ≥1 mm tumour-free resection margin.

Overall survival was defined as the time from resection of the tumour to death. Lymph node ratio is calculated with the number of affected lymph nodes divided by the total number of lymph nodes removed. In the pathological report, the latest (8th) edition of the TNM classification of the AJCC was used.

### Statistical analysis

IBM SPSS Statistics 28.0 (IBM, New York, USA) and R (release 4.3.0) were used for statistical analyses. Survival analyses were performed with the log-rank test and depicted as Kaplan–Meier curves with the number at risk. An adjusted Cox proportional hazard model with overall survival as a dependent variable was employed for multivariate analysis of prognostic factors with 95 per cent c.i. For correlation analysis, Spearman’s correlation coefficient was used. The model of the binomial probability law was used for the calculation to detect at least one lymph node metastasis with a probability of 95 per cent. It was calculated according to the following mathematical formula: *P* = 1 − (1 − lymph node ratio)^examined lymph nodes^, as described previously^15^. Continuous variables were expressed as median and minimum–maximum. Categorical variables were expressed as frequency and percentage and compared with Chi^2^-test/Fisher’s exact test. All tests were two-sided, and *P* < 0.05 was considered to indicate statistical significance.

## Results

### Demographic and histopathological characteristics

Overall, 466 patients who underwent upfront pancreatic resection due to pancreatic ductal adenocarcinoma at the two hospitals were included in the study. Overall, 309 (66.3 per cent) patients received pancreaticoduodenectomy, 84 (18.0 per cent) patients distal pancreatectomy and 73 (15.7 per cent) patients total pancreatectomy for macroscopically complete (R0/R1) tumour resection. The median number of ELN and the median number of positive LN were 23 (i.q.r. 15) and 2 (i.q.r. 5) in pancreaticoduodenectomy, 17 (i.q.r. 12) and 1 (i.q.r. 3) in distal pancreatectomy and 25 (i.q.r. 15) and 2 (i.q.r. 4) in total pancreatectomy. Overall, 125 (27 per cent) patients had no histological evidence of lymph node metastasis (N0 status), whereas in 186 (40 per cent) patients 1–3 positive LN were detected (N1 status), and in 155 (33 per cent) patients more than 3 LN had tumour metastasis (N2 status). The median follow-up of the study cohort was 62.9 months (i.q.r. 46.3) for surviving patients and 16.8 months (i.q.r. 19.3) for deceased patients. The median overall survival of all patients was 22.7 months (i.q.r. 39.1). In patients without lymph node metastasis (N0), the median survival was 52.3 months (i.q.r. 110.4), whereas in patients with lymph node metastasis the survival was significantly worse with 19.8 months (N1) (i.q.r. 26.8) and 16.5 months (N2) (i.q.r. 21.2) (*P* < 0.001). The demographic, clinical and pathological characteristics of all patients are outlined in *Table 1* (stratified by type of resection) and in *Table 2* (stratified by lymph node status). The differences in the study cohorts between centres are shown in *Table 3*. The survival curves according to the lymph node yield did not differ among centres (*Fig. S1*).

**Table 1:** Lymph node status for different types of resection

Parameter	Total	Pancreaticoduodenectomy	Distal pancreatectomy	Total pancreatectomy	*P*
	466 (100%)	309 (66.3%)	84 (18.0%)	73 (15.7%)	
No. of examined lymph nodes, median (min-max)	22 (2–85)	23 (8–69)	17 (2–45)	25 (11–85)	<0.01
No. of positive lymph nodes, median (min-max)	2 (1–25)	2 (1–23)	1 (1–9)	2 (1–25)	0.03
Lymph node ratio, median	0.09	0.10	0.06	0.06	0.11
**Lymph node staging**					
N0	125 (26.8)	79 (25.6)	29 (34.5)	17 (23.3)	0.07
N1	186 (39.9)	117 (37.8)	38 (45.3)	31 (42.5)	
N2	155 (33.3)	113 (36.6)	17 (20.2)	25 (34.2)	

Values are n (%) unless otherwise stated. N1, 1–3 positive lymph nodes; N2, ≥4 positive lymph nodes; lymph node ratio = positive lymph nodes/examined lymph nodes.

**Table 2:** Patient characteristics stratified by lymph node status

Parameter	Total	N0	N1	N2	*P*
	466 (100%)	125 (27%)	186 (40%)	155 (33%)	
**Sex**					
Male	255 (54.7)	65 (52.0)	100 (53.8)	90 (58.1)	0.56
Female	211 (45.3)	60 (48.0)	86 (46.2)	65 (41.9)	
Age (years), median (min-max)	69.3 (32–93)	70.4 (34–93)	69.1 (32–90)	68.5 (35–87)	0.51
**Type of resection**					
Pancreaticoduodenectomy	309 (66.3)	79 (63.2)	117 (62.9)	113 (72.9)	0.057
Distal pancreatectomy	84 (18.0)	29 (23.2)	38 (20.4)	17 (11.0)	
Total pancreatectomy	73 (15.7)	17 (13.6)	31 (16.7)	25 (16.1)	
**T stage (AJCC, 8th edition)**					
T1	61 (13.1)	32 (25.6)	20 (10.7)	9 (5.8)	< 0.001
T2	276 (59.2)	73 (58.4)	121 (65.1)	82 (52.9)	
T3	105 (22.5)	18 (14.4)	38 (20.4)	49 (31.6)	
T4	24 (5.2)	2 (1.6)	7 (3.8)	15 (9.7)	
Tumour size, median (min-max)	32 (1–190)	30 (1–190)	30 (10–110)	35 (10–150)	< 0.001
**Tumour grading**					
1	48 (10.3)	15 (12.0)	19 (10.2)	14 (9.0)	0.61
2	238 (51.1)	66 (52.8)	95 (51.1)	77 (49.7)	
3	170 (36.5)	42 (33.6)	70 (37.6)	58 (37.5)	
4	3 (0.6)	0 (0.0)	2 (1.1)	1 (0.6)	
Unknown	2 (0.4)	2 (1.6)	0 (0.0)	5 (3.2)	
**Resection margin**					
0	153 (32.8)	67 (53.6)	55 (29.6)	31 (20.0)	< 0.001
1	295 (63.3)	54 (43.2)	121 (65.0)	120 (77.4)	
Unknown	18 (3.9)	4 (3.2)	10 (5.4)	4 (2.6)	
**Adjuvant treatment**					
Yes	356 (76.4)	97 (77.6)	140 (75.3)	119 (76.8)	0.94
No	59 (12.7)	14 (11.2)	26 (14.0)	19 (12.3)	
Unknown	51 (10.9)	14 (11.2)	20 (10.8)	17 (11.0)	
Overall survival (months), median	22.7	52.3	19.8	16.5	< 0.001

Values are n (%) unless otherwise stated. T1, size ≤ 2 cm; T2, size ≤4 cm; T3, size >4 cm; T4, infiltration of coeliac trunk, A. hepatica or A. mesenterica sup. R1, distance of the tumour < 1 mm to the resection margin.

**Table 3:** Centre-specific characteristics of the study cohort

Parameter	Total	Munich	Lyon	*P*
	466 (100%)	353 (75.8%)	113 (24.2%)	
No. of examined lymph nodes, median (min-max)	22 (2–85)	22 (5–85)	22(2–47)	0.44
No. of positive lymph nodes, median (min-max)	2 (1–25)	2 (0–25)	3 (0–22)	0.023
Lymph node ratio, median	0.09	0.077	0.135	0.005
**Lymph node status**				
N0	125 (26.8)	103 (29.2)	22 (19.5)	0.12
N1	186 (39.9)	136 (38.5)	50 (44.2)	
N2	155 (33.3)	114 (32.3)	41 (36.3)	
**Type of resection**				
Pancreaticoduodenectomy	309 (66.3)	241 (68.3)	68 (60.2)	0.25
Distal pancreatectomy	84 (18.0)	61 (17.3)	23 (20.4)	
Total pancreatectomy	73 (15.7)	51 (14.4)	22 (19.4)	
Overall survival (months), median	22.7	20.3 (1.9–157.6)	23.1 (3.3–128)	0.47

Values are n (%) unless otherwise stated.

### Influence of the number of examined lymph nodes on nodal staging

**Fig. 2:**
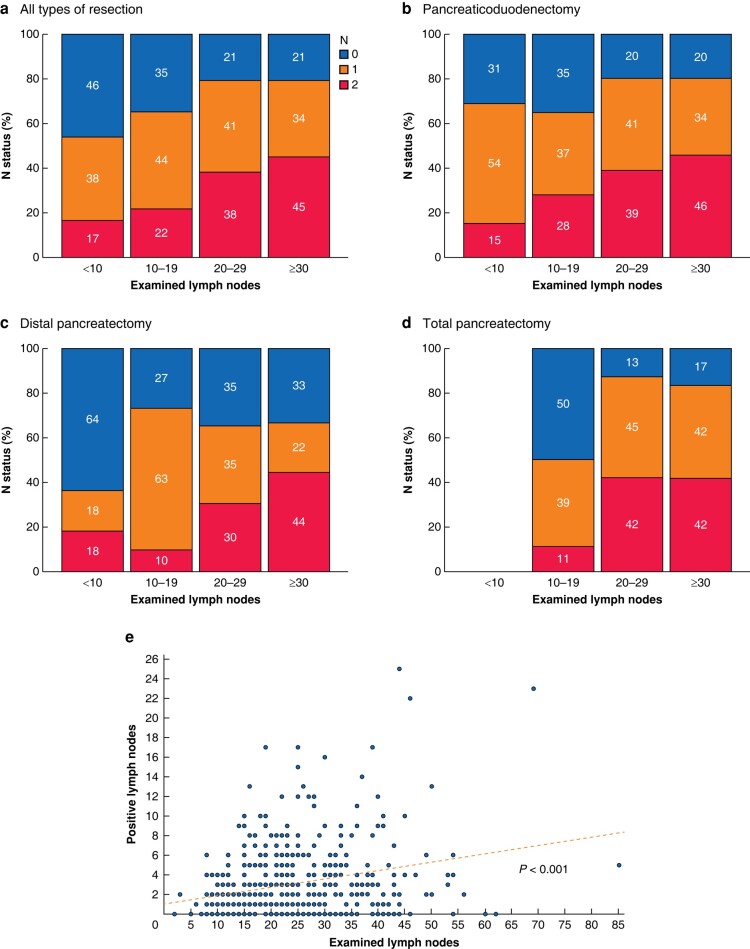
Distribution of the number of examined lymph nodes (ELN) on nodal status Patients were categorized into four groups according to the number of ELN (I: <10 ELN; II: 10-19 ELN; III: 20-29 ELN; IV: ≥30 ELN) and analysed for each type of resection (**a–d**). Correlation analysis of examined lymph nodes and positive lymph nodes with Spearman's correlation (**e**).

The study cohort was classified into four different groups according to the amount of ELN (group I: < 10 ELN; group II: 10–19 ELN; group III: 20–29 ELN; group IV: ≥ 30 ELN). Concerning all patients, the proportion of lymph node positive status (N1 and N2) increased with increasing amounts of ELN. In patients with ELN <10, 56 per cent (*n* = 13) showed lymph node metastasis (38 per cent (*n* = 9) N1 and 17 per cent (*n* = 4) N2), in patients with 10–19 ELN 66 per cent (*n* = 108) (44 per cent (*n* = 72) N1 and 22 per cent (*n* = 36) N2) compared to 79 per cent (*n* = 116) (41 per cent (*n* = 60) N1 and 38 per cent (*n* = 56) N2) when 20–29 LN were examined, and 79 per cent (*n* = 104) (34 per cent (*n* = 45) N1 and 45 per cent (*n* = 59) N2) when at least 30 LN were histologically examined (*Fig. 2a*).

After splitting the cohort based on the type of resection the trend towards a higher proportion of lymph node metastasis with increasing number of ELN persisted (*Fig. 2b–d*).

Amount of ELN correlated significantly with positive LN detected (*P* < 0.001) (*Fig. 2e*), but not with tumour size (*P* = 0.187).

### Calculation of minimum number of examined lymph nodes for adequate nodal staging

The binominal probability law was used to calculate the minimum number of ELN needed to detect at least one positive lymph node. The lymph node ratio was 0.09 in all patients, 0.10 in patients after pancreaticoduodenectomy, 0.06 after distal pancreatectomy and 0.06 after total pancreatectomy. To detect at least one positive lymph node with a probability of 95 per cent, a minimum ELN of 21 was needed in all patients, 20 in pancreaticoduodenectomy, 25 in distal pancreatectomy and 22 for total pancreatectomy.

### Impact of nodal staging on survival

**Fig. 3:**
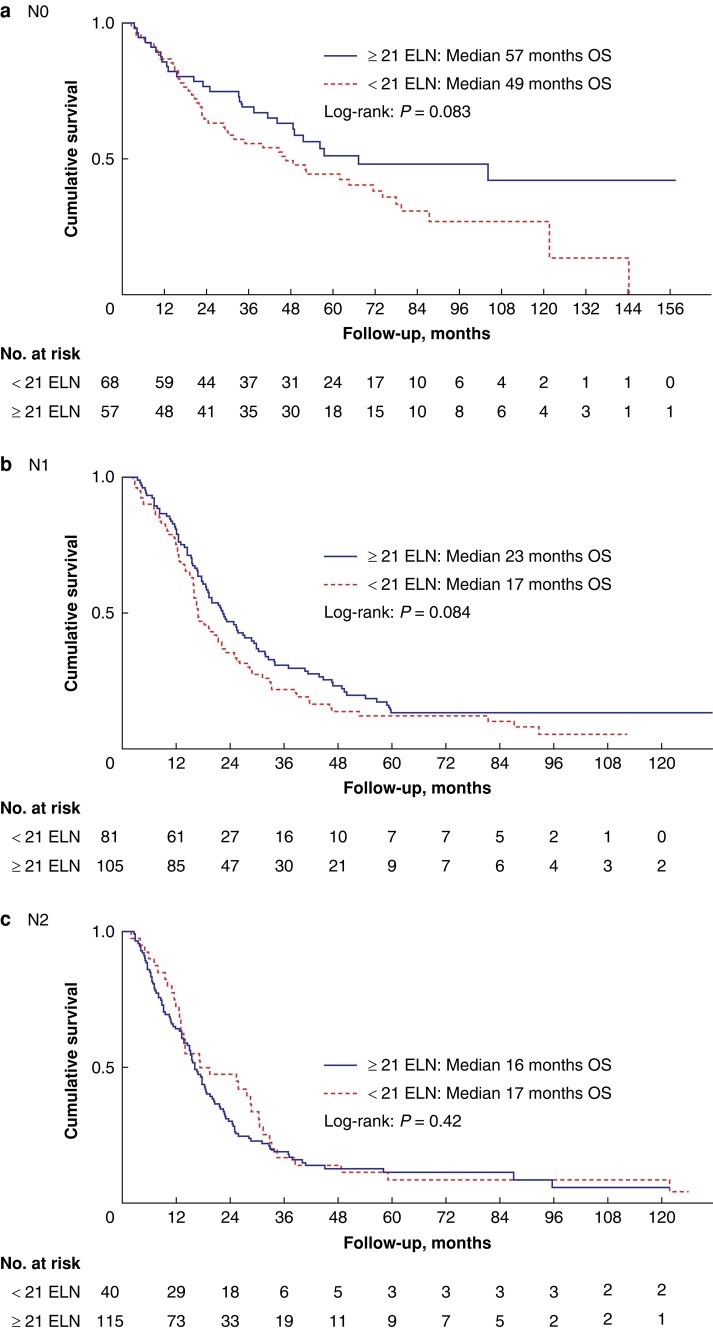
Overall survival (OS) of all patients stratified by nodal status according to the value of 21 examined lymph nodes (ELN) (a–c)

To find a cut-off value that prevents misclassification, the calculated minimum number of ELN needed to detect one positive LN was analysed for its impact on survival. In all patients, the median overall survival did not show significant differences when <21 LN were examined compared to ELN of ≥21 (*Fig. 3a–c*) (*P* = 0.10).

Univariate and multivariate analysis with Cox regression model was performed including the most affecting factors^17–19^ for overall survival in pancreatic cancer to analyse the impact of 21 ELN on survival (*Tables 4*, *5*). After adjusting with the other variables, Cox regression analysis showed that the examination of <21 lymph nodes was a significant negative predictor for overall survival (HR 1.27, 95 per cent c.i. 1.02–1.58, *P* = 0.034). The best cut-off value of ELN was determined when ELN had no significant impact on survival. Interestingly, the examination of <22 LN was not significant in the multivariate analysis (HR 1.23, 95 per cent c.i. 0.99–1.60, *P* = 0.061). The most influencing factor for worsening overall survival in this cohort was lymph node involvement with N1 status (HR 2.14, 95 per cent c.i. (1.59–2.86), *P* < 0.001) and N2 status (HR 2.57, 95 per cent c.i. (1.88–3.51), *P* < 0.001). The multivariate analysis was also performed separately for each lymph node status (N0, N1 and N2) without any differences (*Table S1*).

**Table 4:** Univariate analysis of prognostic factors for overall survival

Variable	HR	95% c.i.	*P*
Sex, female	0.854	(0694–1.050)	0.13
Age (years)	1.008	(1.008–1.018)	0.12
**T stage (AJCC, 8th edition)**			
T1	1.0 (reference)		–
T2	2.29	(1.59–3.31)	< 0.001
T3	3.27	(2.19–4.89)	< 0.001
T4	5.03	(2.94–8.60)	< 0.001
**Lymph node status**			
N0	1.0 (reference)		–
N1	2.35	(0.177–3.12)	< 0.001
N2	2.96	(2.21–3.95)	< 0.001
**Tumour grade**			
G1	1.0 (reference)		–
G2	1.39	(0.96–2.02)	0.07
G3	1.93	(1.32–2.83)	< 0.001
**Resection margin**			
R0	1.0 (reference)		–
R1	1.55	(1.23–1.95)	< 0.001

**Table 5:** Multivariate analysis of prognostic factors for overall survival

Analysis: <21 examined lymph nodes				Analysis: <22 examined lymph nodes			
Variable	HR	95% c.i.	*P*	Variable	HR	95% c.i.	*P*
T-stage: T3/T4	1.52	1.19–1.93	<0.001	T-stage: T3/T4	1.50	1.18–1.91	<0.001
Grading: G3/G4	1.44	1.16–1.79	<0.001	Grading: G3/G4	1.44	1.15–1.79	<0.001
R1-status*	1.26	0.99–1.60	0.051	R1-status*	1.27	0.99–1.53	0.051
**Lymph node status**				**Lymph node status**			
N1	2.16	1.61–2.89	<0.001	N1	2.14	1.59–2.86	<0.001
N2	2.57	1.88–3.51	<0.001	N2	2.57	1.88–3.51	<0.001
<21 ELN	1.27	1.02–1.58	0.034	<22 ELN	1.23	0.99–1.60	0.061

*Defined as R0 ≥ 1 mm tumour-free resection margin. Adjusted Cox proportional hazard model, HR with 95 per cent c.i., examined lymph nodes (ELN).

## Discussion

The present study highlighted the importance of adequate lymph node dissection and examination in pancreatic cancer surgery. One of the most important findings was that with increasing number of ELN, the proportion of LN metastasis increased. From these results, it was concluded that patients with a low number of ELN are often falsely classified in the N-category, which leads to ‘understaging’ of these patients. In other words, patients classified as N0 are in fact N1, and N1 patients are actually N2 if more LN were removed and examined. A valid prognosis for these patients could have been achieved by more accurate and extensive lymph node sampling. Because lymphadenectomy is supposed to be a standardized procedure, the pathological lymph node examination was probably insufficient. Misclassification can either be caused by inadequate lymphadenectomy, or by insufficient histological examination. Further categorization and analysis of misclassification would require histologic re-evaluation of the resected specimen.

Multivariate analysis of the main factors influencing survival (including tumour size^18^, surgical resection margin^17^, pathological grading^19^ and lymph nodes status^5^) was used to evaluate a cut-off value of ELN to prevent misclassification. In the adjusted regression model, the examination of <21 LN was a negative predictor for overall survival. Therefore examining at least 21 LN in pancreatic resection prevents possible misclassification and can be seen as the best cut-off point.

Lymph node status is also dependent on the experience of the surgeon. Higher median LN count has been reported in high-volume centres^20^. Pathological assessment and identification of lymph nodes in the resected tissue is necessary and it depends on the pathologist’s expertise. Larger LN are less likely to be missed, as a lymph node about 4 mm in diameter can be palpable and easily identified. Complete embedding of the fatty tissue in addition can help to increase the number of ELN and was shown to influence nodal staging in oesophageal adenocarcinoma^21^. The amount of resected LN may also be influenced by the patient’s individual constitution. Autopsy studies revealed different numbers of regional LN with individual differences in LN amount in gastric cancer^22,23^. It is also known that overweight and high BMI hampers lymphadenectomy in gastric cancer^24^.

Several studies addressed the role of lymph node examination on survival in pancreatic cancer. In this study the highest median examined lymph node count was collected and all different types of pancreatic resection were considered. Hellan and coworkers^12^ postulated in a study with 1915 node-negative patients that more than 10 LN should be examined for improved survival, but the median number of ELN in the study was 7, which is relatively low compared to 22 LN in the present cohort. A study by Slidell *et al.*^13^ with 1507 node-negative patients proposed that more than 12 LN should be examined to avoid misclassification with a median ELN of 7 LN. Both studies included patients from 1988 to 2003 where quality of lymphadenectomy and histopathological assessment were different than today. Tomlinson *et al.*^14^ concluded from a study with 3505 patients that the examination of at least 15 LN is optimal for accurate staging in pancreatic cancer but reported only a median ELN of 7.

The statistical calculation of the minimum number of ELN needed to detect at least one possible LN using the binominal law has been carried out before. For pancreaticoduodenectomy, Vuarnesson *et al*.^15^ calculated 16 required ELN and Malleo *et al.*^11^ 20 LN for distal pancreatectomy. Taking into account that previous studies have a lower median ELN, the present paper will serve as a reference for the minimum needed number of 21 LN for adequate staging. The European Society for Medical Oncology (ESMO) suggests the examination of ≥15 LN in pancreaticoduodenectomy as recommended by the International Study Group on Pancreatic Surgery (ISGPS)^25^. In the recently updated German S3-Guideline on the treatment of pancreatic cancer (AWMF), the minimum number of ELN needed to classify as N0 was increased to 12 LN^26^.

The study had several limitations. Due to its retrospective nature, the study was limited by lack of some clinical data and by the impossibility of reassessing all the histological specimens with specific focus on lymph node examination. No data were available on disease-free survival, CA19-9 levels and peri- and vascular invasion. Patients with neoadjuvant treatment were excluded from the final analysis. Neoadjuvant therapy was not the standard treatment during the study period and contained several different treatment protocols.

After splitting the study cohort according to different resection types, there were no significant survival differences detected, probably due to the small number of subgroups.

Standardized lymph node resection and systematic, careful pathological assessment are needed for accurate staging and achieving the best outcome for PDAC patients. From these results, at least a minimum of 21 lymph nodes are needed for adequate examination in all PDAC patients to avoid misclassification of N0 and N1 status by surgical or pathological reasons. This number has to be evaluated in different study cohorts to confirm the results. A uniform recommendation for a minimum need of ELN can improve the comparability of patients and thereby improve survival in the long term.

## Supplementary Material

zrad125_Supplementary_Data

## Data Availability

The data presented in this study are available on request from the corresponding author.
