# Supplementary material for: Lymph node examination and survival in resected pancreatic ductal adenocarcinoma: retrospective study
Source: BJS Open. 2024 Jan 25;8(1):zrad125. doi: 10.1093/bjsopen/zrad125 (PMC10810280; doi:10.1093/bjsopen/zrad125)
Supplement: zrad125_Supplementary_Data [file zrad125_supplementary_data.docx]

**Lymph node examination and survival in resected pancreatic ductal adenocarcinoma:**

**retrospective study**

Ruediger Goess^1,3,4^, Carsten Jäger^1,3,4^, Julie Perinel^2^, Ilaria Pergolini^1,3,4^, Elke Demir^1,3,4^, Okan Safak^1,3,4^, Florian Scheufele^1,3,4^, Stephan Schorn^1,3,4^, Alexander Muckenhuber^5^, Mustapha Adham^2^, Alexander Novotny^1^, Güralp O. Ceyhan^6^, Helmut Friess^1,3,4^, Ihsan Ekin Demir ^1,3,4,6,7^

^1^Department of Surgery, Klinikum rechts der Isar, Technical University of Munich, School of Medicine, Munich, Germany

^2^Department of Digestive Surgery, E. Herriot Hospital, Hospices civils de Lyon, Lyon, France

^3^German Cancer Consortium (DKTK), Partner Site Munich, Germany

^4^CRC 1321 Modelling and Targeting Pancreatic Cancer

^5^Institute of Pathology, Klinikum rechts der Isar, Technical University of Munich, School of Medicine, Munich, Germany

^6^Department of General Surgery, HPB-Unit, School of Medicine, Acibadem Mehmet Ali Aydinlar University, Istanbul, Turkey

^7^Else Kröner Clinician Scientist Professor for Translational Pancreatic Surgery

**Corresponding author:** Ihsan Ekin Demir, MD, Department of Surgery, Klinikum rechts der Isar, Technical University of Munich, Ismaninger Str. 22, D-81675 München, Germany, Tel.: +49 89 4140 5868, Fax: +49 89 4140 4870, E-Mail: ekin.demir@tum.de

**Supplementary Materials - Index**

| **Supplementary Methods** |  |
| --- | --- |
| none |  |
| **Supplementary Results** |  |
| none |  |
| **Supplementary Appendixes** |  |
| none |  |
| **Supplementary Figures and Tables** |  |
| Supplementary Figure 1 | *pag. 6* |
| Supplementary Table 1 | *pag. 7* |
| **References** | *pag. 12-13* |
|  |  |

**Supplementary Methods**

**Supplementary Results**

**Supplementary AppendixesSupplementary Figures and Tables**

**Supplementary Figure 1:** Overall survival of all patients (N0/N1/N2) using the identified cut-off value of 21 examined lymph nodes (ELN) separately for each centre: Munich cohort (A) and Lyon cohort (B)

**
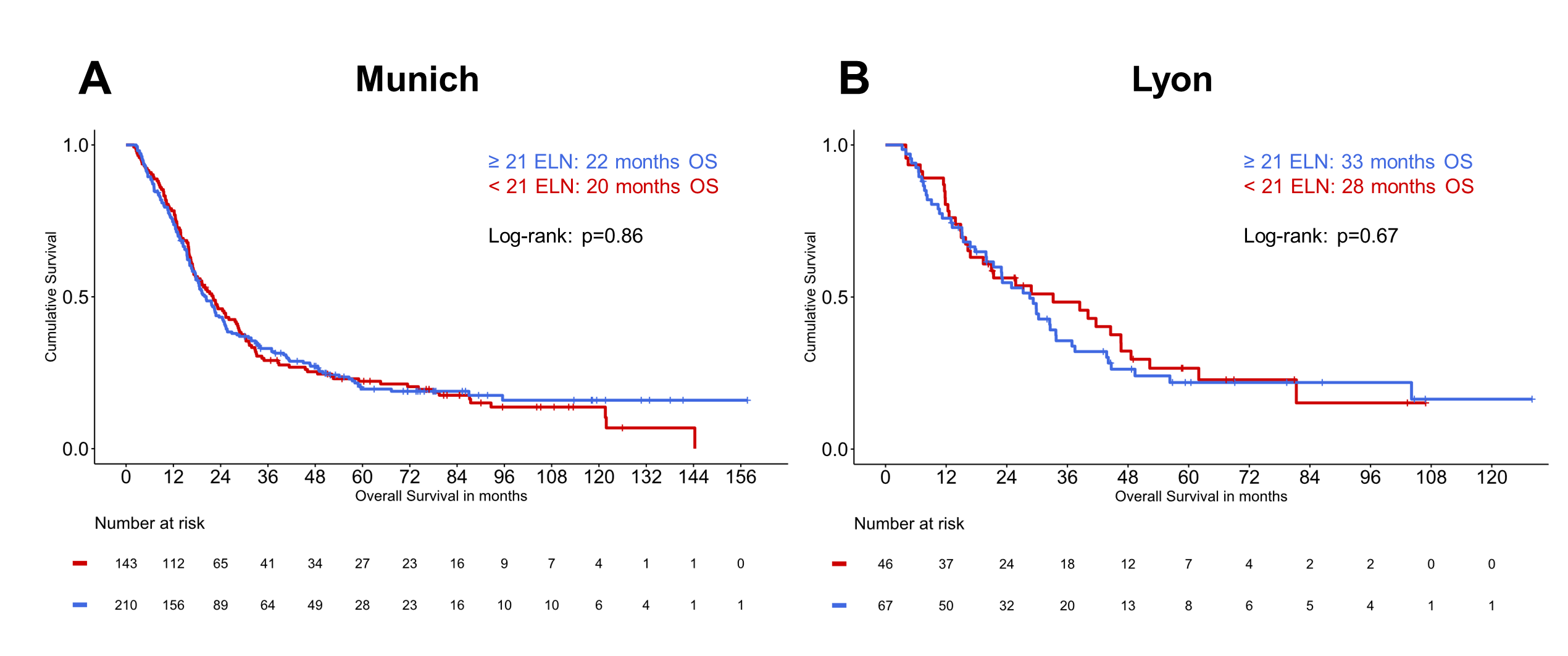
**

**Supplementary Table 1:**

| **Supplementary Table 1. Multivariate analysis of prognostic factors for overall survival separated by LN status** | | | | | | | | | | |  |
| --- | --- | --- | --- | --- | --- | --- | --- | --- | --- | --- | --- |
|  |  |  |  |  |  |  |  |  |  |  |  |
|  | **Only N0 patients** | | |  | **Only N1 patients** | | |  | **Only N2 patients** | | |
| **Variable** | **HR** | **95% CI** | **p-value** |  | **HR** | **95% CI** | **p-value** |  | **HR** | **95% CI** | **p-value** |
| T-stage: T3/T4 | 2.54 | (1.45 - 4.45) | 0.001 |  | 1.52 | (1.03 - 2.25) | 0.033 |  | 1.18 | (0.82 - 1.71) | 0.360 |
| Grading: G3/G4 | 1.48 | (0.91 - 2.42) | 0.110 |  | 1.44 | (1.02 - 2.04) | 0.039 |  | 1.40 | (0.97 - 2.02) | 0.068 |
| R1-status* | 1.12 | (0.69 - 1.79) | 0.636 |  | 1.20 | (0.83 - 1.72) | 0.324 |  | 1.62 | (1.03 - 2.56) | 0.037 |
| < 21 ELN | 1.50 | (0.92 - 2.46) | 0.102 |  | 1.26 | (0.91 - 1.75) | 0.157 |  | 0.99 | (0.67 - 1.49) | 0.991 |
|  |  |  |  |  |  |  |  |  |  |  |  |
| *defined as R0 ≥ 1mm tumor free resection margin | | | |  |  |  |  |  |  |  |  |
| Adjusted Cox Proportional Hazard Model, Hazard ratios (HR) with 95% confidence intervals (CI), examined lymph node (ELN) | | | | | | | | | | |  |

**References**

1. Mizrahi JD, Surana R, Valle JW, Shroff RT. Pancreatic cancer. Lancet. 2020;395:2008-20.

2. Rhim AD, Mirek ET, Aiello NM, Maitra A, Bailey JM, McAllister F, et al. EMT and dissemination precede pancreatic tumor formation. Cell. 2012;148:349-61.

3. Malleo G, Maggino L, Capelli P, Gulino F, Segattini S, Scarpa A, et al. Reappraisal of Nodal Staging and Study of Lymph Node Station Involvement in Pancreaticoduodenectomy with the Standard International Study Group of Pancreatic Surgery Definition of Lymphadenectomy for Cancer. J Am Coll Surg. 2015;221:367-79 e4.

4. Pawlik TM, Gleisner AL, Cameron JL, Winter JM, Assumpcao L, Lillemoe KD, et al. Prognostic relevance of lymph node ratio following pancreaticoduodenectomy for pancreatic cancer. Surgery. 2007;141:610-8.

5. House MG, Gonen M, Jarnagin WR, D'Angelica M, DeMatteo RP, Fong Y, et al. Prognostic significance of pathologic nodal status in patients with resected pancreatic cancer. J Gastrointest Surg. 2007;11:1549-55.

6. Cameron JL, Riall TS, Coleman J, Belcher KA. One thousand consecutive pancreaticoduodenectomies. Ann Surg. 2006;244:10-5.

7. Strobel O, Hinz U, Gluth A, Hank T, Hackert T, Bergmann F, et al. Pancreatic adenocarcinoma: number of positive nodes allows to distinguish several N categories. Ann Surg. 2015;261:961-9.

8. Farnell MB, Pearson RK, Sarr MG, DiMagno EP, Burgart LJ, Dahl TR, et al. A prospective randomized trial comparing standard pancreatoduodenectomy with pancreatoduodenectomy with extended lymphadenectomy in resectable pancreatic head adenocarcinoma. Surgery. 2005;138:618-28; discussion 28-30.

9. Riall TS, Cameron JL, Lillemoe KD, Campbell KA, Sauter PK, Coleman J, et al. Pancreaticoduodenectomy with or without distal gastrectomy and extended retroperitoneal lymphadenectomy for periampullary adenocarcinoma--part 3: update on 5-year survival. J Gastrointest Surg. 2005;9:1191-204; discussion 204-6.

10. Tol JA, Gouma DJ, Bassi C, Dervenis C, Montorsi M, Adham M, et al. Definition of a standard lymphadenectomy in surgery for pancreatic ductal adenocarcinoma: a consensus statement by the International Study Group on Pancreatic Surgery (ISGPS). Surgery. 2014;156:591-600.

11. Malleo G, Maggino L, Ferrone CR, Marchegiani G, Mino-Kenudson M, Capelli P, et al. Number of Examined Lymph Nodes and Nodal Status Assessment in Distal Pancreatectomy for Body/Tail Ductal Adenocarcinoma. Ann Surg. 2019;270:1138-46.

12. Hellan M, Sun CL, Artinyan A, Mojica-Manosa P, Bhatia S, Ellenhorn JD, et al. The impact of lymph node number on survival in patients with lymph node-negative pancreatic cancer. Pancreas. 2008;37:19-24.

13. Slidell MB, Chang DC, Cameron JL, Wolfgang C, Herman JM, Schulick RD, et al. Impact of total lymph node count and lymph node ratio on staging and survival after pancreatectomy for pancreatic adenocarcinoma: a large, population-based analysis. Ann Surg Oncol. 2008;15:165-74.

14. Tomlinson JS, Jain S, Bentrem DJ, Sekeris EG, Maggard MA, Hines OJ, et al. Accuracy of staging node-negative pancreas cancer: a potential quality measure. Arch Surg. 2007;142:767-23; discussion 73-4.

15. Vuarnesson H, Lupinacci RM, Semoun O, Svrcek M, Julie C, Balladur P, et al. Number of examined lymph nodes and nodal status assessment in pancreaticoduodenectomy for pancreatic adenocarcinoma. Eur J Surg Oncol. 2013;39:1116-21.

16. Warschkow R, Widmann B, Beutner U, Marti L, Steffen T, Schiesser M, et al. The More the Better-Lower Rate of Stage Migration and Better Survival in Patients With Retrieval of 20 or More Regional Lymph Nodes in Pancreatic Cancer: A Population-Based Propensity Score Matched and Trend SEER Analysis. Pancreas. 2017;46:648-57.

17. Demir IE, Jager C, Schlitter AM, Konukiewitz B, Stecher L, Schorn S, et al. R0 Versus R1 Resection Matters after Pancreaticoduodenectomy, and Less after Distal or Total Pancreatectomy for Pancreatic Cancer. Ann Surg. 2018;268:1058-68.

18. Moon HJ, An JY, Heo JS, Choi SH, Joh JW, Kim YI. Predicting survival after surgical resection for pancreatic ductal adenocarcinoma. Pancreas. 2006;32:37-43.

19. Sohn TA, Yeo CJ, Cameron JL, Koniaris L, Kaushal S, Abrams RA, et al. Resected adenocarcinoma of the pancreas-616 patients: results, outcomes, and prognostic indicators. J Gastrointest Surg. 2000;4:567-79.

20. Bilimoria KY, Talamonti MS, Wayne JD, Tomlinson JS, Stewart AK, Winchester DP, et al. Effect of hospital type and volume on lymph node evaluation for gastric and pancreatic cancer. Arch Surg. 2008;143:671-8; discussion 8.

21. Quaas A, Schloesser H, Fuchs H, Zander T, Arolt C, Scheel AH, et al. Improved Tissue Processing in Esophageal Adenocarcinoma After Ivor Lewis Esophagectomy Allows Histological Analysis of All Surgically Removed Lymph Nodes with Significant Effects on Nodal UICC Stages. Ann Surg Oncol. 2021;28:3975-82.

22. Herbella FAM, Lourenco LG, Bonini AL, Schlottmann F, Patti MG. Anatomical analysis of gastric lymph nodes in cancer-free individuals. Clin Anat. 2019;32:9-12.

23. Wagner PK, Ramaswamy A, Ruschoff J, Schmitz-Moormann P, Rothmund M. Lymph node counts in the upper abdomen: anatomical basis for lymphadenectomy in gastric cancer. Br J Surg. 1991;78:825-7.

24. Dhar DK, Kubota H, Tachibana M, Kotoh T, Tabara H, Masunaga R, et al. Body mass index determines the success of lymph node dissection and predicts the outcome of gastric carcinoma patients. Oncology. 2000;59:18-23.

25. Ducreux M, Cuhna AS, Caramella C, Hollebecque A, Burtin P, Goere D, et al. Cancer of the pancreas: ESMO Clinical Practice Guidelines for diagnosis, treatment and follow-up. Ann Oncol. 2015;26 Suppl 5:v56-68.

26. Seufferlein T, Mayerle J, Bock S, Brunner T, Ettrich TJ, Grenacher L, et al. S3-Leitlinie zum exokrinen Pankreaskarzinom - Langversion 2.0. Z Gastroenterol. 2022;60:e812-e909.
